# RETRACTED: Sibuh et al. The Emerging Role of Epigenetics in Metabolism and Endocrinology. *Biology* 2023, *12*, 256

**DOI:** 10.3390/biology14060705

**Published:** 2025-06-16

**Authors:** Belay Zeleke Sibuh, Sameer Quazi, Hrithika Panday, Ritika Parashar, Niraj Kumar Jha, Runjhun Mathur, Saurabh Kumar Jha, Pankaj Taneja, Abhimanyu Kumar Jha

**Affiliations:** 1 Department of Biotechnology, School of Engineering and Technology, Sharda University, Knowledge Park III, Greater Noida 201310, India; 2 GenLab Biosolutions Private Limited, Bangalore 560043, India; 3 Department of Biomedical Sciences, School of Life Sciences, Anglia Ruskin University, Cambridge CB1 1PT, UK; 4 Clinical Bioinformatics, School of Health Sciences, The University of Manchester, Manchester M13 9P, UK; 5 SCAMT Institute, ITMO University, St. Petersburg 197101, Russia; 6 School of Bioengineering & Biosciences, Lovely Professional University, Phagwara 144411, India; 7 Department of Biotechnology, School of Applied & Life Sciences (SALS), Uttaranchal University, Dehradun 248007, India; 8 Department of Biotechnology Engineering and Food Technology, Chandigarh University, Mohali 140413, India

The Editorial Office of *Biology* retracts the paper entitled “*The Emerging Role of Epigenetics in Metabolism and Endocrinology*” [1], cited above.

Following publication, concerns were brought to the attention of the publisher regarding the authenticity of the authorship of this article [1].

Adhering to our complaint procedure, an investigation was conducted by the Editorial Office and Editorial Board. Following communications with the authorship group, the Editorial Board was unable to verify the identity, contribution, or affiliations of a number of the authors listed in this manuscript, nor could the origins of the study be confirmed. As a consequence, the Editorial Board has lost confidence in the integrity of the findings and has decided to retract this publication [1] as per MDPI’s retraction policy (https://www.mdpi.com/ethics#_bookmark30).

This retraction was approved by the Editors-in-Chief of the journal *Biology*.

The authors did not agree to this retraction.
